# Intra-cavitary fluid resulted from caesarean section but not isthmocele compromised clinical pregnancy after IVF/ICSI treatment

**DOI:** 10.1007/s00404-022-06436-0

**Published:** 2022-03-26

**Authors:** Meihong Cai, Xinyi Pan, Wei Xia, Xiaoyan Liang, Xing Yang

**Affiliations:** 1 Department of Reproductive Medicine Centre, The Sixth Affiliated Hospital of Sun Yat-Sen University, Guangzhou, Guangdong China; 2 Department of Reproductive Medicine Centre, Guangzhou First People’s Hospital, School of Medicine, South China University of Technology, Guangzhou, Guangdong China

**Keywords:** Caesarean section (CS), Intra-cavitary fluid (ICF), Isthmocele, Clinical pregnancy, Assisted reproductive technologies, Secondary infertility

## Abstract

The aim of this study was to explore whether the presence of intra-cavitary fluid (ICF) influences the pregnancy outcomes of patients with caesarean section (CS) in embryo transfer cycles. A total of 8494 transferred cycles of 4924 women were enrolled in this retrospective study and separated into three subgroups by previous delivery method and the presence of intra-cavity fluid, a caesarean group with ICF (CS-ICF, *n* = 649), a caesarean group without ICF (CS-noICF, *n* = 3207), and the remaining 4638 cycles without ICF were included in the vaginal delivered group (VD, *n* = 4638). Baseline characteristics and clinical outcome were compared. Propensity score matching (PSM) was conducted to adjust confounding factors between groups. Patients in the CS-ICF group were of younger age (36.49 ± 4.19 vs 37.34 ± 4.25, 37.32 ± 4.86, *P* < 0.001), had better ovary reserve, and had more blastocyst transferred compared with the CS-noICF and VD groups. However, cycles in the CS-ICF group achieved unsatisfactory clinical pregnancy outcomes. PSM analysis for comparability and differences in clinical outcomes still existed. The clinical pregnancy rate was significantly lower in the CS-ICF group than in the CS-noICF group (35.1% vs 41.7% for CS-noICF group, 48.1% for VD group, *P* < 0.001). Subgroup analysis of fresh embryo transferred cycles, the differences in clinical outcomes disappeared after PSM analysis, while the clinical pregnancy rate was still lowest among the three matched groups of FET cycles (36.4% vs 50.3% for VD group, *P* < 0.001). The presence of intra-cavitary fluid (ICF), but not necessarily the isthmocele, significantly compromises the clinical pregnancy rate in patients with previous CS undergoing IVF/ICSI treatment.

## Introduction

Caesarean section (CS) rates are increasing worldwide [1, 2]. In China, the rate increased from 28.8% in 2008 to 36.7% in 2018, especially for women who had received ART treatment [3]. The scar is considered to have an impact on subsequent pregnancies. Initial delivery by CS leads to a lower pregnancy rate compared to that after a previous vaginal delivery (VD) [4, 5]. Women with a previous history of CS are 10% less likely to have a subsequent pregnancy or birth compared to women who deliver by VD [6]. A growing number of women, mostly with previous caesarean deliveries and/or advanced age, are seeking assisted reproduction technology since the introduction of the 2-child policy in China [3], and since many delayed childbirth due to having full-time occupation or seeking higher education [7]. The increasing CS rate has stimulated attention to the CS scar and the women’s clinical outcomes after undergoing in vitro fertilization (IVF)/intracytoplasmic sperm injection (ICSI).

Previous research has been directed towards the impact of the CS scar and the isthmocele. Isthmocele is a defect which measures 1–2 mm or seen as a hypoechoic triangle in the anterior myometrium at the site of lower segment cesarian section scar [8]. Prior research has examined the association of isthmocele and pregnancy outcome among patients undergoing IVF [9]. This study instead focused on how the scar impairs endometrium receptivity and the prevention of future treatment for intra-cavitary fluid (ICF). This is one of the first studies to examine the impact of previous delivery mode and the CS-associated ICF.

With the development of ultrasound technology, clinicians are paying increasing attention to the CS defect and have confirmed a high incidence rate; 56% of women with a previous history of CS exhibited a niche and associated postmenstrual spotting as reported by Vaate et al. [10]. Previous studies revealed a significant uncompromised outcome of patients with a history of CS. It was reported that the existence of a CS scar may impact embryo implantation and clinical pregnancy outcome compared to vaginal delivery (VD) in a retrospective study [11]. Lawrenz et al. also reported the relationship between ICF and uncompromised clinical outcomes on frozen embryo transfer (FET) cycles, which were unaffected when ICF cases were excluded [9]. The potential causes of the lower pregnancy rates in patients with a history of CS still remain to be investigated.

ICF during IVF has attracted clinicians’ concern, especially in patients with hydrosalpinx, polycystic ovary, uterine cavity infection, and endometriosis [12], and can cause unreceptive endometrium due to inflammation, mechanical interference, flushing, or gene expression changes. The nature of the effusion may be blood, mucus, purulent, or transparent, and cannot be classified by an ultrasound exam. Studies on patients with CS history focus mostly on the scar defect and report difficult embryo transfers due to the isthmocele [9]. A systematic review concluded that if difficult transfers can be converted into easy ones, pregnancy outcomes can be improved [13]. ICF in patients with a CS history may better explain uncompromised outcomes.

The decline in pregnancy rate is related to many factors, including ovarian reserve, embryo quality, endometrium receptivity, and cross-talk between embryo and endometrium. Ovarian reserve indices such as anti-Müllerian hormone (AMH) and antral follicle count (AFC) are not affected by delivery mode [14]. Most of the patients can achieve good-quality embryos as reported in Chinese patients [15]. For patients with a history of CS, endometrial problems can account for most of the failures in fresh cycles of IVF. Moreover, the reported incidence of ICF may reach 40% [9]. We hypothesize that the presence of ICF may play an important role in reducing the endometrium receptivity and may further affect pregnancy outcomes. The goal of this study was to explore whether the presence of ICF influences the pregnancy outcomes of patients with caesarean section (CS) in embryo transfer cycles.

## Materials and methods

### Population

All the embryo transfer cycles of patients with at least one previous birth were enrolled; moreover, patients with an endometrium thinner than 7 mm on the day of embryo transfer or with adenomyosis were excluded. There were 8494 cycles that underwent IVF/ICSI treatment from January 2014 to July 2020 in a university-based hospital. According to the previous delivery method, the 8494 cycles included were separated into two groups, including 3685 cycles with a history of caesarean section and 4638 cycles with a history of vaginal delivery. ICF was diagnosed by ultrasound monitoring, referring to uterine cavity separation over 3 mm before and on the day of triggering. Patients with a history of caesarean section were further separated into a caesarean group with ICF (CS-ICF group, *n* = 649) and a caesarean group without ICF (CS-noICF group, *n* = 3207). The data were extracted from medical databases, including baseline characteristics, controlled ovarian hyperstimulation procedures, and pregnancy outcomes.

### Controlled ovarian hyperstimulation

For controlled ovarian stimulation, the following protocols were used.

Long protocol: 1.875 mg of leuproreline acetate (3.75 mg Diphereline@; Ipsen, France) was administered in the mid-luteal phase of the previous menstrual period. Fourteen days later, ultrasound and hormonal levels of follicle-stimulating hormone (FSH), Luteinizing hormone (LH), and Estradiol (E2) were examined, and recombinant FSH (Gonal-F, Merck Serono, Germany) or high-purity urofollitropin (Lishenbao, Lizhu, China) was prescribed daily according patients’ individual baseline characteristics.

Antagonist protocol: exogenous gonadotropin (Gn) was started daily on day 2 of the menstrual period. Four days later, antagonist (Cetrorelix, Merck Serono, Germany) was added for preventing early ovulation, and ultrasound and hormonal levels were evaluated and adjusted accordingly.

Ultralong protocol: 3.75 mg of leuproreline acetate was administered on day 2 of the menstrual period once for 1–3 cycles, and 28 days later, ovarian stimulation was started by Gn. Ultrasound and hormonal levels were evaluated and adjusted accordingly.

Other unconventional protocols: modified natural cycle with a small amount of Gn or a mini-dose protocol with a small dose of Gn was administered if the patient has low ovarian reserves.

When two follicles were larger than 18 mm or three follicles were larger than 17 mm, 10,000 IU of human chorionic gonadotropin was administered intra-muscularly for triggering, and oocyte retrieval was performed after 36 h.

### Endometrium preparation protocol

For frozen–thawed embryo transfer cycles, the following protocols for endometrium preparation were used.

Natural cycle or ovulation stimulation cycle: patients with regular menstrual cycles were monitored throughout a natural FET cycle. Anovulatory patients may choose drugs to induce ovulation, undergoing a daily ultrasonographic scan from the 10th–12th days of the menstrual cycle until the dominant follicle disappeared. The day of ovulation was considered D0. The transfer was performed 3 or 5 days later accordingly.

Hormone replacement therapy cycle: patients with irregular menstrual cycles were treated with estradiol (Progynova, Bayer, Leverkusen, Germany), with a dosage of 2 mg orally, twice a day, for approximately 10–14 days, which was adjusted according to endometrial thickness. When the endometrial thickness was more than 8 mm, progesterone was added, and this day was considered D0. The transfer was performed after 3 or 5 days accordingly.

### Luteal support and embryo transfer

Progesterone (Duphaston, Abbott, The Netherlands) was given 20 mg twice a day on D0 (oocyte retrieval) orally. The fertilization method and the number of embryos transferred were decided according to the clinicians’ experience based on a standard procedure. Embryo transfer was conducted with 1–2 cleavage embryos on D3 or 1–2 or blastocysts on D5 after oocyte retrieval when patients had good endometrium conditions and the width of the ICF was no more than 3 mm. Cleavage stage embryo evaluation was based on the Peter scoring system [16] and Blastocysts were assessed by the Gardner scoring system [17]. Cleavage embryo grades 1 or 2 with at least five blastomeres were considered transferrable embryos and grades 1 or 2 with 6–10 blastomeres were considered good-quality embryos; blastocysts graded 3BB and above were considered good-quality blastocysts.

### Statistical analyses

#### Missing information

Some patients were missing some basic data, such as AMH, FSH, and total gonadotropin and days, but the proportion was less than 10%. These values were analysed as missing value.

### Data analysis

The measured data were described by mean ± standard deviation (SD), compared by ANOVA for normally distributed count data and compared by Kruskal–Wallis test for non-normally distributed count data. The count data were described as rate (%) and were compared by Chi-square test. To further analyse the effect of ICF on the clinical pregnancy of patients with a previous history of CS, binary logistic regression was conducted. We adjusted for age, BMI, AFC, treatment protocol, and embryonic development stage by propensity score matching (PSM).

All statistical analyses were performed using SPSS 22.0 (IBM), and results with a two-tailed *α* level of 0.05 were considered to be significant.

## Results

### Baseline characteristics and clinical outcomes of the enrolled patients

A total of 8494 transferred cycles, both fresh embryo transferred cycles and FET cycles, of 4924 women were enrolled and separated into three subgroups by previous delivery method and the presence of ICF into a CS-ICF group (*n* = 649), a CS-noICF group (*n* = 3207) and a VD group (*n* = 4638). Table 1 shows the details of demographic data of these enrolled cycles. The average age of this study population was approximately 37 years. Patients in the CS-ICF group were of younger age (36.49 ± 4.19 vs 37.34 ± 4.25, 37.32 ± 4.86, *P* < 0.001), and had better ovary reserves (AFC 10.20 ± 7.31 vs 8.96 ± 5.97, 8.93 ± 6.35, *P* < 0.001) and more blastocysts transferred (72.7% vs 62.4%, 47.0%, *P* < 0.001) than those in the CS-noICF and VD groups. The CS-ICF group experienced more FET cycles than the other two groups (80.6% vs 64.0% for CS-noICF group, 59.1% for VD group, *P* < 0.001). In addition, the treatment protocols were significantly different among the three groups.

**Table 1:** Baseline characteristics of three groups, CS-ICF, CS-noICF, and VD groups

	CS-ICF group (*n* = 649)	CS-noICF group (*n* = 3207)	VD group (*n* = 4638)	*P* value
Age (years)	36.49 ± 4.19	37.34 ± 4.25	37.32 ± 4.86	< 0.001
BMI	22.71 ± 6.19	23.15 ± 3.02	22.62 ± 3.21	< 0.001
AFC (*n*)	10.20 ± 7.31	8.96 ± 5.97	8.93 ± 6.35	< 0.001
Cycle type	/	/	/	< 0.001
Fresh embryo transferred cycle	126 (19.4%)	1156 (36.0%)	1899 (40.9%)	/
FET cycle	523 (80.6%)	2051 (64.0%)	2739 (59.1%)	/
Treatment protocol	/	/	/	< 0.001
Conventional protocol	113 (17.4%)	1038 (32.4%)	1642 (35.4%)	/
Micro-stimulation protocol	13 (2.0%)	118 (3.7%)	257 (5.5%)	/
Ovulatory cycle or natural cycle	236 (36.4%)	776 (24.2%)	948 (20.4%)	/
Artificial cycle	287 (44.2%)	1275 (39.8%)	1791 (38.6%)	/
No. of embryos transferred (*n*)	1.41 ± 0.51	1.45 ± 0.50	1.64 ± 0.50	< 0.001
1 embryos	384 (59.2%)	1778 (55.4%)	1730 (37.3%)	< 0.001
2 embryos	261 (40.2%)	1419 (44.2%)	2873 (61.9%)	/
3 embryos	4 (0.6%)	10 (0.3%)	35 (0.6%)	/
Embryo stage	/	/	/	< 0.001
Cleavage stage	177 (27.3%)	1205 (37.6%)	2456 (53.0%)	/
Blastosphere stage	472 (72.7%)	2002 (62.4%)	2182 (47.0%)	/

All data are presented as mean ± SD. *P* < 0.05 is considered statistically significant

*BMI* body mass index, *FSH* follicle-stimulating hormone, *AMH* anti-Müllerian hormone, *AFC* antral follicle count, *No.* number, *Gn* gonadotropin, *2PN* 2 pronuclei

However, there was a tendency that patients in the CS-ICF group had undergone more years of infertility (5.45 ± 3.54, *P* < 0.001) and worse clinical outcomes, as shown in Table 2. The biochemical pregnancy rates (*P* < 0.001), clinical pregnancy rates (*P* < 0.001), implantation rates (*P* < 0.001), and live birth rates (*P* < 0.001) differed significantly across the three groups, and were lowest in the CS-ICF group. The ectopic pregnancy rates were comparable among the three groups (*P* = 0.233), while the miscarriage rate was lowest in the CS-ICF group (*P* = 0.002).

**Table 2:** Clinical outcome of three groups, CS-ICF, CS-noICF, and VD groups

	CS-ICF group (*n* = 649)	CS-noICF group (*n* = 3207)	VD group (*n* = 4638)	*P* value
Duration of subfertility	5.45 ± 3.54	4.16 ± 3.22	4.57 ± 3.80	< 0.001
Biochemical pregnancy rate, *n* (%)	248 (38.2%)	1352 (42.2%)	2208(47.6%)	< 0.001
Clinical pregnancy rate, *n* (%)	227 (35.0%)	1209 (37.7%)	2022 (43.6%)	< 0.001
Implantation rate, % (*n*)	26.3% (241/918)	28.9% (1343/4646)	31.2% (2369/7581)	< 0.001
Ectopic pregnancy rate, *n* (%)	6 (0.9%)	13 (0.4%)	24 (0.5%)	0.233
Miscarriage rate, *n* (%)	42 (7.0%)	326 (10.7%)	507 (11.6%)	0.002
Live birth rate	135 (20.8%)	758 (23.6%)	1271 (27.4%)* (1vs2, 3; 2vs3)	< 0.001
Gestation at delivery	37.20 ± 2.37	37.57 ± 1.96	37.92 ± 1.92	< 0.001

*P* < 0.05 is considered statistically significant

### Baseline characteristics and clinical outcomes of the enrolled patients with PSM analysis

Upon further analysis with 1:2:2 PSM, there were 3090 cycles included and the baseline characteristics such as age, BMI, and ovarian reserves of three groups were comparable (Table 3). The proportions of cycle types and treatment protocols were equal among three groups. However, differences in clinical outcomes still existed. Patients in the CS-ICF group still suffered from a longer duration of infertility (5.42 ± 3.55, *P* < 0.001). The biochemical pregnancy rates (38.0% vs 46.0% for the CS-noICF group, 51.6% for the VD group, *P* < 0.001), clinical pregnancy rates (35.1% vs 48.1% for the VD group, *P* < 0.001), and live birth rates (21.0% vs 29.5% for the VD group, *P* < 0.05) differed significantly across the three groups, and were lowest in the CS with ICF group. Moreover, the ectopic pregnancy rates were slightly higher in the CS-ICF group and VD groups than in the CS-noICF group (*P* = 0.023), while the miscarriage rate was lowest in the CS-ICF group (*P* < 0.001) (Table 4).

**Table 3:** Baseline characteristics of three groups, CS-ICF, CS-noICF, and VD groups after 1:2:2 PSM analysis

	CS-ICF group (*n* = 618)	CS-noICF group (*n* = 1236)	VD group (*n* = 1236)	*P* value
Age (year)	36.54 ± 4.23	36.59 ± 4.44	36.78 ± 4.89	0.469
BMI	22.57 ± 2.77	22.52 ± 2.58	22.51 ± 2.68	0.879
AFC (*n*)	10.09 ± 7.07	10.08 ± 6.52	9.87 ± 6.58	0.683
Cycle type	/	/	/	0.674
Fresh embryo transferred cycle	124	248	232	/
FET cycle	494	988	1004	/
Treatment protocol	/	/	/	0.360
Conventional protocol	111 (18.0%)	217 (17.6%)	212 (17.2%)	/
Micro-stimulation protocol	13 (2.1%)	31 (2.5%)	20 (1.6%)	/
Ovulatory cycle	21 8 (35.3%)	436 (35.3%)	403 (32.6%)	/
Artificial cycle	276 (44.7%)	552 (44.7%)	601 (48.6%)	/
No. of embryos transferred (*n*)	1.42 ± 0.51	1.40 ± 0.49	1.41 ± 0.49	0.581
Embryo stage	/	/	/	0.727
Cleavage stage	171 (27.7%)	342 (27.7%)	321 (26.0%)	/
Blastosphere stage	447 (72.3%)	894 (72.3%)	915 (74.0%)	/

All data are presented as mean ± SD. *P* < 0.05 is considered statistically significant

*BMI* body mass index, *FSH* follicle-stimulating hormone, *AMH* anti-Müllerian hormone, *AFC* antral follicle count, *No.* number, *Gn* gonadotropin, *2PN* 2 pronuclei

**Table 4:** Clinical outcome of three groups, CS-ICF, CS-noICF, and VD groups after 1:2:2 PSM analysis

	CS-ICF group (*n* = 618)	CS-noICF group (*n* = 1236)	VD group (*n* = 1236)	*P* value
Duration of subfertility	5.42 ± 3.55	4.16 ± 3.09	4.48 ± 3.71	< 0.001
Biochemical pregnancy, *n* (%)	23 (38.0%)	568 (46.0%)	638 (51.6%)	< 0.001
Clinical pregnancy rate, *n* (%)	217 (35.1%)	516 (41.7%)	594 (48.1%)	< 0.001
Ectopic pregnancy rate, *n* (%)	6 (1.0%)	2 (0.2%)	12 (1.0%)	0.023
Miscarriage rate, *n* (%)	42 (6.8%)	163 (13.2%)	143 (11.6%)	< 0.001
Live birth rate	130 (21.0%)	324 (26.2%)	365 (29.5%)	< 0.001
Missing	39 (6.3%)	27 (2.2%)	74 (6.0%)	< 0.001
Gestation at delivery	37.19 ± 2.41	37.65 ± 1.83	37.99 ± 2.10	< 0.001

*P* < 0.05 is considered statistically significant

### Subgroup analysis

In the subgroup of fresh transferred cycles, there were 601 cycles enrolled in matched groups. With paired baseline characteristics, they were comparable in terms of age, BMI, AFC, embryonic development stage, and number of embryos transferred. As expected, the CS-ICF group was found to experience the lowest biochemical pregnancy rate compared with the CS-noICF and VD groups (*P* = 0.021). However, differences in the duration of subfertility (*P* = 0.061), clinical pregnancy rate (*P* = 0.058), miscarriage rate (*P* = 0.069), ectopic pregnancy rate (*P* = 0.419), and the live birth rate (*P* = 0.094) were not significantly different among the three paired groups (Table 5).

**Table 5:** Clinical outcome of three groups, CS-ICF group, CS-noICF, and VD group, for subgroup of fresh embryo transferred cycles after 1:2:2 PSM analysis

	CS-ICF group (*n* = 121)	CS-noICF group (*n* = 240)	VD group (*n* = 240)	*P* value
Duration of subfertility	5.15 ± 4.05	4.14 ± 3.42	4.54 ± 4.11	0.061
Biochemical pregnancy, *n* (%)	38 (31.4%)	111 (46.2%)	106 (44.2%)	0.021
Clinical pregnancy rate, *n* (%)	35 (28.9%)	94 (39.2%)	99 (41.3%)	0.066
Ectopic pregnancy rate, *n* (%)	1 (0.8%)	1 (0.4%)	0 (0.0%)	0.419
Miscarriage rate, *n* (%)	6 (5.0%)	22 (9.2%)	30 (12.5%)	0.069
Live birth rate	20 (16.5%)	64 (26.7%)	54 (22.5%)	0.094
Missing value	8 (6.6%)	7 (2.9%)	15 (6.3%)	0.161
Gestation at delivery	36.16 ± 3.42	37.65 ± 1.82	37.72 ± 1.73	0.015

*P* < 0.05 is considered statistically significant

Interestingly, in the subgroup of FET cycles, with paired baseline characteristics, resulting in comparable age, BMI, AFC, embryonic development stage, and number of embryos transferred, the clinical pregnancy rate of cycles in the CS-ICF group was significantly lower than those of the CS-noICF and VD groups, while the duration of subfertility was much longer. There were significant decreases in terms of biochemical pregnancy, miscarriage, and live birth rates in the CS-ICF group compared with the CS-noICF and VD groups. There was no significance difference in the ectopic pregnancy rate among the three groups (Table 6).

**Table 6:** Clinical outcome of three groups, CS-ICF, CS-noICF, and VD groups, for subgroup of FET cycles after 1:2:2 PSM analysis

	CS-ICF group (*n* = 492)	CS-noICF group (*n* = 981)	VD group (*n* = 981)	*P* value
Duration of subfertility	5.49 ± 3.42	4.08 ± 2.97	4.58 ± 3.70	< 0.001
Biochemical pregnancy, *n* (%)	194 (39.4%)	423 (43.1%)	539 (54.9%)	< 0.001
Clinical pregnancy rate, *n* (%)	179 (36.4%)	378 (38.5%)	493 (50.3%)	< 0.001
Ectopic pregnancy rate, *n* (%)	5 (1.0%)	2 (0.2%)	5 (0.5%)	0.108
Miscarriage rate, *n* (%)	35 (7.1%)	99 (10.1%)	139 (14.2%)	< 0.001
Live birth rate	109 (22.2%)	255 (26.0%)	288 (29.4%)	0.011
Missing value	30 (6.1%)	22 (2.2%)	61 (6.2%)	< 0.001
Gestation at delivery	37.36 ± 2.17	37.40 ± 2.08	37.78 ± 2.24	0.080

*P* < 0.05 is considered statistically significant

## Discussion

### Main findings

We have shown that there was a significant decrease in clinical outcomes for patients in the CS-ICF group compared with the CS-noICF and VD groups, which may be explained by Salker’s research outcome of impaired decidualization [18]. These differences still existed when baseline characteristics were matched after PSM. Excessive intra-uterine fluid has been found to result in changes in the expression of genes related to endometrium receptivity, such as integrin *α* and *β* in mice [19]. Most patients with a CS history may experience prolonged menstrual periods, or a weakened barrier of cervical mucus, which may cause uterine inflammation and impaired decidualization. Another explanation may lie in the presence of the scar, which may change the myometrial contractility and influence embryo implantation. The scar may destroy the continuity of the muscle fibers, and those with severe impacts may result in ICF and further extremely early pregnancy loss [20].

Our data show a negative effect between ICF and clinical pregnancy rather than an impact based on merely the presence of a scar and the clinical pregnancy rate. Most studies have suggested that women with ICF have low implantation and pregnancy rates in IVF cycles [12, 21, 22]. Our data confirm that CS history is not the major cause of uncompromised clinical outcome during IVF cycle; however, the presence of ICF may explain the phenomenon. We were not concerned about the association of ICF and the scar defect, as a previous study conducted by Lawrenz et al. had indicated similar reproductive outcomes between patients with and without an isthmocele when ICF was cured [9]. The fluid which may be caused by the history of caesarean section and further influence of microbial flora changes could be of major importance. Additionally, we have conducted another prospective study which analysed the differences in uterus microbial flora between women with post-caesarean section (CS) scar diverticulum and women after vaginal delivery, exploring the correlation between differentially expressed microbial flora and inflammation and found that the disrupted uterus microbiota composition in women with CS may be closely associated with local inflammation [23].

There were fewer embryos transferred in the CS-ICF group than in the CS-noICF and VD groups according to clinicians’ tendency to employ a single blastocyst transfer strategy with smaller residue muscular thickness. Though it was not recorded with objective data, the proportions of one embryo transferred and one blastocyst transferred were highest among the three groups (59.2% of one embryo and 72.2% of one blastocyst). However, the ectopic pregnancy rates were similar among the three groups. This is inconsistent with the previous studies among patients with a history of CS, as it has been reported that CS is a risk factor for ectopic pregnancies [24, 25]. The difference could be explained by the high proportion of blastocyst transfers, as a single blastocyst transfer may be a good intervention implemented to reduce the ectopic pregnancy rate compared with cleavage stage embryo transfer [26]. The potential effects of CS or ICF on ectopic pregnancy may be masked by the high ratio of blastocyst transfers.

With subgroup analysis, interesting findings can be seen between fresh cycles and frozen embryo transfer cycles. It seems the impacts of ICF disappeared in fresh embryo transfer cycles (a difference was shown only in biochemical pregnancy), while the adverse impact of ICF was still apparent in frozen embryo transfer cycles. However, there were a downward trend in clinical pregnancy and live birth rates, as the rates were lowest in the CS-ICF group almost 10% lower than that in the CS-noICF group. The fact that differences did not reach statistical significance may be due to the reduction in sample size after matching. The number of frozen thaw embryo transfer cycles enrolled for analysis was more than four times that of fresh cycles, which may be explained by clinicians’ prioritizing frozen strategy when the presence of ICF was obvious. However, the adverse impact of ICF still existed in FET cycles, since it was difficult to treat under the current level of medical technology, and we failed to analyse the treatment of isthmocele or ICF in the following FET cycle as a retrospective study.

ICF may be cause by many factors, such as hydrosalpinges, polycystic ovarian disease, subclinical uterine infections, and endometriosis [12, 21]. Our data show that the proportion of ICF associated with hydrosalpinx or OHSS was less than 5%, as fluid associated with hydrosalpinx [21] and PCOS [27] has been confirmed to impact clinical outcomes, and most such patients underwent a frozen protocol in our centre. The potential cause of ICF may be explained by endometriosis, a finding that is supported by two other studies in non-tubal factor patients [28, 29]. The possible mechanism is stenosis of the cervical canal and formation of isthmocele, which may induce fluid accumulation mechanically or menstrual dripping and can cause difficulty in embryo transfer.

Changes in the endometrial microenvironment caused by the presence of ICF may be the main cause of implantation failure. The occurrence of ICF during IVF is generally considered to be related to fallopian tube factors, hormone levels in superphysiological states and related changes in the uterine microenvironment. In addition, the CS isthmocele may cause iatrogenic adenomyosis, which may result in toxic environment and impair endometrial receptivity and cervicouterine microbiota [30]. ICF with a history of CS may result in effusion from the ectopic endometrium in the scar, which has been confirmed pathologically in 28% of hysterectomy specimens of 51 patients in Morris’ study [31]. Gurbuz et al. [29] provided an indirect evidence by describing a non-invasive isthmocele treatment in which patients were treated with gonadotropin-releasing hormone agonist for 3 months before hormone replacement treatment in FET cycles, which results in 25% of live birth.

We compared clinical outcomes among patients who had a history of CS with or with ICF, as well as patients who underwent vaginal delivery and were convinced that the clinical pregnancy rate was lower in the CS-ICF group than in the CS-noICF and VD groups. Vissers et al. [32] also reported an impact on clinical pregnancy and implantation of CS, but they did not perform further analysis of the scar and its associated effects. In the recent study by Lawrenz et al. [9], an association between ICF and uncompromised clinical outcomes was mentioned, and the researchers conducted a detailed analysis of the possible factors of the production of effusion. He et al. [33] have reviewed the treatment options for endometrial cavity fluid (ECF), including expectant treatment, postponing embryo transfer, transvaginal sonographic ECF aspiration, and other subsidiary modification. Another study concluded that a subgroup of patients with symptomatic isthmocele may benefit from hysteroscopic isthmoplasty, which can help confirm the effect of ICF on pregnancy from another perspective [34].

### Limitations and strengths

A potential criticism of this study is that we did not record the association of ICF with scar defect, as our focus was on the presence of ICF rather than the defect. Our study also has another limitation as we could not determine the degree of separation and the time of ICF development, since no data were collected on the amount of ICF; however, this does not affect our analysis, as in our clinical routine management, if the ICF was more than 3 mm, we adopt an embryo freezing strategy rather than fresh embryo transfer, which can be supported by Andersen et al.’s study [35]. We also acknowledge that another limitation may lie in the cancellation cycles, which may include additional patients with ICF. In addition, these data do not allow us to determine the initial factors of the decrease in live births following CS in patients with or without ICF, as the study was retrospective in design, and we were unable to obtain inflammation information for the ICF, which remains for further research in the future. With these limitations, our strength lies in the group design, comparing the presence of ICF as well as delivery mode. In addition, our study contains the largest sample size in a retrospective study of this subject to date.

### Further study

Our study proposed an added analysis for the uncompromised clinical outcomes of patients with a history of CS. We cannot say definitely that with the presence of a CS scar has an impact on clinical outcomes with the mere presence of ICF, but we can say that the effect of the scar on the endometrium environment can lead to an unfavorable outcome, but not the scar itself. The association between ICF and delivery mode is novel, and verifying whether the same association applies to general patients with or without scar defects and whether the association is affected by the amount of effusion would be interesting. At this time, we have no way to clarify the nature of the effusion, as the study was retrospective in design. In future research, we may focus on the bacterial analysis of effusion to explore the direct mechanism and propose improved protocols by way of a large prospective cohort study. Further study is being undertaken to ascertain why a history of CS is associated with fewer clinical pregnancies.

### Implications of our findings

Even with the limitations, our results are still of great clinical significance. With the increasing CS rate worldwide, more infertile women are seeking to become pregnant with a scar on their uterus. Many studies have confirmed an uncompromised clinical outcome in patients with a history of CS and reported an even lower pregnancy rate when a niche was mentioned [9, 27, 34, 36, 37]. Previous studies agree that there is an impact in clinical outcomes, though some of them focus on isthmocele, and they describe blood or mucus on catheter [38]. ICF may be a new, effective, treatable factor that should be paid attention to achieve better pregnancy outcomes in patients with a history of CS.

## Conclusion

It is the presence of ICF and not necessarily that of the isthmocele that significantly compromised the clinical pregnancy rate in patients with CS. CS interferes with the integrity of the uterine wall, ICF may be accompanied by inflammatory cytokines and potentially pathogenic bacteria, which further disrupt the endometrial receptivity. A large prospective cohort study is required to better understand the potential mechanism and explore effective therapeutic strategies.

## Data Availability

The data that support the findings of the study are available from the corresponding author upon reasonable request.
